# Attitudes Towards Detection and Management of Hepatic Metastases of Colorectal Origin: A Second Look

**DOI:** 10.1155/1994/64894

**Published:** 1994

**Authors:** David J. Bruinvels, L. Maurits de Brauw, Job Kievit, J. Dik F. Habbema, Cornelis J. H. van de Velde

**Affiliations:** 1 Medical Decision Making Unit University of Leiden Leiden The Netherlands; 2 Department of Surgery University Hospital Leiden Leiden The Netherlands; 3 Department of Surgery Free University Hospital Amsterdam The Netherlands; 4 Center for Clinical Decision Sciences Department of Public Health Erasmus University Rotterdam The Netherlands; 5 Medical Decision Making Unit Building 43, Room 1.002 University Hospital Leiden P.O. Box. 9600 Leiden 2300 RC The Netherlands

## Abstract

In the present study we undertook an international postal survey to assess the current attitudes towards the detection and management of hepatic metastases in colorectal cancer patients, who have been operated on with curative intent. Results of this survey were compared to results of an earlier survey, held in 1985. Both surveys indicate that there is no consensus on the follow-up of patients at risk of hepatic metastases. Especially the interpretation of unexplained rises in carcinoembryonic antigen (CEA) levels leads to much controversy. Only 37% of the hospitals performing liver surgery were willing to perform second-look laparotomies based on CEA only. Also there is no agreement on the maximum number of liver metastases that will justify partial liver resection for cure. Clearly, there is a need for prospective randomized trials on which a more rational policy regarding hepatic metastases in colorectal cancer patients can be based.


*HPB Surgery*, 1994, Vol. 8, pp. 115–122
Reprints available directly from the publisher
Photocopying permitted by license only

© 1994 Harwood Academic Publishers GmbH
Printed in Malaysia

### Attitudes Towards Detection and Management of Hepatic Metastases of Colorectal Origin: A Second Look

DAVID J. BRUINVELS[1,2], L. MAURITS DE BRAUW[3], JOB KIEVIT*[1,2], J. DIK F. HABBEMA[4], and CORNELIS J. H. VAN DE VELDE[2]

[1] Medical Decision Making Unit, University of Leiden, Leiden, The Netherlands
[2] Department of Surgery, University Hospital Leiden, Leiden, The Netherlands
[3] Department of Surgery, Free University Hospital, Amsterdam, The Netherlands
[4] Center for Clinical Decision Sciences / Department of Public Health, Erasmus University, Rotterdam, The Netherlands

*(Received 25 January 1994)*

In the present study we undertook an international postal survey to assess the current attitudes towards the detection and management of hepatic metastases in colorectal cancer patients, who have been operated on with curative intent. Results of this survey were compared to results of an earlier survey, held in 1985. Both surveys indicate that there is no consensus on the follow-up of patients at risk of hepatic metastases. Especially the interpretation of unexplained rises in carcinoembryonic antigen (CEA) levels leads to much controversy. Only 37% of the hospitals performing liver surgery were willing to perform second-look laparotomies based on CEA only. Also there is no agreement on the maximum number of liver metastases that will justify partial liver resection for cure. Clearly, there is a need for prospective randomized trials on which a more rational policy regarding hepatic metastases in colorectal cancer patients can be based.

KEY WORDS: Questionnaires colonic neoplasms rectal neoplasms liver neoplasms, secondary liver neoplasms, surgery

### INTRODUCTION

Colorectal cancer is the second leading cause of death from cancer[1]. Although 75% of colorectal cancer patients will have a primary surgical resection for cure, nearly half of all patients with colorectal cancer still die of metastatic tumor[2]. Resection of liver metastases and adjuvant chemotherapy are currently the most promising developments in the treatment of colorectal cancer patients.

Because there exists worldwide controversy among clinicians regarding optimal diagnostic and therapeutic policies, we held in 1985 a postal survey of 290 hospitals in 13 Western countries[3]. The response rate was 98%, which is exceptionally high for a postal survey. Nearly all hospitals in this survey used a systematic follow-up aimed at detecting liver metastases in colorectal cancer patients operated for cure and performed liver surgery when necessary. Approximately 75% of the hospitals treated patients with unresectable liver metastases with chemotherapy, and 33% of the hospitals used adjuvant chemotherapy following curative resection of liver metastases.

In 1992 we held, encouraged by our earlier results, a second postal survey. We developed an extended version of the 1985 questionnaire, which we sent to 1955 clinicians in 77 countries. Results of this survey may give a more detailed and complete view on current attitudes toward hepatic metastases.

* *Correspondence to:* J. Kievit, M.D., Ph.D., Medical Decision Making Unit, Building 43, Room 1.002, University Hospital Leiden, P.O. Box. 9600, 2300 RC Leiden, The Netherlands, Telephone: NL-71276780, Fax: NL-71225520.

### MATERIALS AND METHODS

In 1992 we sent a postal questionnaire to all 1955 members of the 'World Association of Hepato-Pancreato-Biliary Surgery'. Addresses were obtained from the 1990 HPB membership registry.

The questionnaire consists of four parts:

(1) Detection of liver metastases by systematic follow-up: Which diagnostic tests were used with which frequency? Were diagnostic second-look laparotomies performed?
(2) Resection of liver metastases: What was the maximum number of metastases considered for radical resection, and were they confined to one or two lobes? How many patients were operated for potentially resectable liver metastases in 1991, and in how many patients were liver metastases actually resected? Which preoperative imaging techniques were used to assess the number of liver metastases? Was the resectability of liver metastases intraoperatively assessed using ultrasound imaging?
(3) Adjuvant chemotherapy following potentially curative resection of colorectal cancer or partial liver resection: Which drugs were administered by which route?
(4) Chemotherapy of unresectable liver metastases: How many patients were treated in 1991? By which route were drugs administered?

A sample of the questionnaire is included in the Appendix.

### RESULTS

### *Response rate*

The 1990 HPB membership registry listed 1955 names and addresses of clinicians involved in the field of hepatic, pancreatic and biliary surgery. Of these 1955 clinicians 903 were listed by their private address and 1052 by their hospital address. The 1052 clinicians listed by their hospital address were employed in 653 different hospitals. Thus, in total, there were 1556 questionnaires sent.

Fifty-three of these questionnaires were returned to us unopened, indicating that at least 3% of the addresses we used were incorrect.

A total of 171 questionnaires were answered and returned to us. The 1992 questionnaire, however, was sent to 77 countries worldwide, in contrast to the 1985 questionnaire, which was sent to 13 Western countries* only. The response rate of the recent survey calculated for these 13 Western countries is 21%.

### *Detection of liver metastases*

In the first section of the 1992 questionnaire we asked clinicians to describe the follow-up they used for colorectal cancer patients operated for cure. Systematic routine follow-up was used in 150 (88%) of the hospitals. Of these hospitals 134 (89%) used follow-up schemes of five years or longer.

Scheduled history taking and physical examinations were used in 144 (96%) hospitals, routine liver biochemistry in 119 (79%), serum tumor markers in 141 (94%), liver ultrasonography in 111 (74%), and computerized tomography (CT) in 65 (43%). The average number of individual diagnostic tests per year are presented in Table 1.

Serum carcinoembryonic ântigen (CEA) assays were used by all 141 hospitals which used tumor markers. Other serum tumor markers also used by these hospital were CA 19-9 (carbohydrate antigen associated with gastrointestinal cancer) in 32 hospitals, AFP (alpha-fetoprotein) in 9 hospitals, TPA (tissue polypeptide antigen) in 4 hospitals, CA 50 (carbodydrate antigen associated with carcinomas) in 3 hospitals, CA 125 (carbohydrate antigen associated with ovarian cancer) in 2 hospitals, CA 195 (carbohydrate antigen associated with gastrointestinal cancer) in 1 hospital, ferritin in 1 hospital, and NCC-ST 439 (monoclonal antibody against gastric cancer cells) also in 1 hospital.

Second-look laparotomies solely based on significant and consecutive rises in CEA-levels were performed in 52 (37%) of the 141 hospitals.

Compared to the 1958 survey, fewer hospitals performed clinical evaluations and liver biochemistry tests

**Table 1** Mean number of diagnostic tests per year used to detect liver metastases

| *Diagnostic test* | *Year of follow-up* | | | | |
|---|---|---|---|---|---|
| | *1st* | *2nd* | *3rd* | *4th* | *5th* |
| Clinical evaluation | 3 | 3 | 2 | 2 | 2 |
| Liver biochemistry | 3 | 2 | 2 | 1 | 1 |
| Tumor markers | 4 | 3 | 2 | 2 | 2 |
| Liver ultrasonography | 2 | 2 | 1 | 1 | 1 |
| Liver CT | 1 | 1 | 1 | 1 | 1 |

* Austria, Belgium, Canada, France, Germany, Israel, Italy, Spain, Sweden, Switzerland, the Netherlands, the United Kingdom (UK), and the United States of America (USA).

**Table 2** Diagnostic tests used to detect liver metastases in 13 Western countries

| *Diagnostic test* | *Percentage of hospitals per country* | | | | | | |
|---|---|---|---|---|---|---|---|
| | *France* | *Germany* | *The Netherlands* | *UK* | *USA and Canada* | *Other* | *All* |
| *1985 Survey* | | | | | | | |
| Clinical evaluation | 100 | 100 | 100 | 91 | 100 | 94 | 98.2 |
| Liver biochemistry | 88 | 95 | 96 | 59 | 90 | 88 | 87.3 |
| Serum CEA | 88 | 100 | 73 | 50 | 97 | 85 | 75.0 |
| Imaging techniques | 84 | 95 | 55 | 41 | 64 | 73 | 64.1 |
| *1992 Survey* | | | | | | | |
| Clinical evaluation | 100 | 100 | 86 | 50 | 75 | 91 | 82.4 |
| Liver biochemistry | 75 | 91 | 50 | 42 | 68 | 85 | 70.6 |
| Serum CEA | 100 | 100 | 79 | 50 | 79 | 97 | 84.3 |
| Imaging techniques | 100 | 100 | 36 | 33 | 61 | 88 | 64.7 |

on a routine basis in 1991 (Table 2). Consequently, serial CEA testing was the most frequently used screening method in colorectal cancer follow-up.

### *Resection of liver metastases*

In the second section of the 1992 questionnaire we asked about the surgical aspects of liver metastases. In 144 hospitals, in which numerical data on colorectal cancer patients were available, liver metastases were detected in 3250 patients in 1991. Potentially resectable liver metastases were never considered for resection in 5 (3%) of the hospitals and were resected only when solitary in28(16%) of the hospitals. In 81 (47%) of the hospitals multiple metastases were resected when they were confined to one lobe, with a maximum of 3 metastases, while 57 (33%) of the hospitals also resected multiple metastases not confined to one lobe, with a maximum of 4 metastases. In 36 (21%) hospitals resectability was assessed without a reference to the number of metastases. In 1991 a total of 1238 (38%) patients had surgery for liver metastases, and in 911 (74%) of these patients liver metastases were actually resected.

When liver metastases were suspected, resectability was assessed using preoperative imaging techniques. Ultrasonography of the abdomen was used by 135 (82%) hospitals, CT (computerized tomography) by 112 (68%) hospitals, CT combined with angiography in 103 (62%) hospitals, MRI (magnetic resonance imaging) by 31 (19%) hospitals, and scintigraphy by 9 (5%) hospitals. The resectability of liver metastases was assessed by intraoperative ultrasonography of the liver in 85 (58%) of the hospitals which performed liver resections in 1991. No increase in intraoperatively diagnosed unresectable recurrences was reported by the hospitals which used this technique.

In comparison to the 1985 survey more hospitals resected multiple bilobar metastases in 1991 (Table 3). The number of liver resections per hospital has also

**Table 3** Criteria for resection of liver metastases in 13 Western countries

| *Preoperative criteria* | *Percentage of hospitals per country* | | | | | | |
|---|---|---|---|---|---|---|---|
| | *France* | *Germany* | *The Netherlands* | *UK* | *USA and Canada* | *Other* | *All* |
| *1985 Survey* | | | | | | | |
| No surgery | 0 | 0 | 18 | 6 | 0 | 3 | 5.3 |
| One solitary metastasis | 20 | 5 | 48 | 47 | 27 | 27 | 32.4 |
| Multiple unilobar metastases | 64 | 37 | 31 | 44 | 41 | 61 | 43.0 |
| Multiple bilobar metastases | 16 | 58 | 3 | 3 | 32 | 9 | 19.4 |
| *1992 Survey* | | | | | | | |
| No surgery | 0 | 0 | 7 | 8 | 4 | 0 | 2.9 |
| One solitary metastasis | 25 | 9 | 0 | 8 | 4 | 21 | 10.8 |
| Multiple unilobar metastases | 25 | 36 | 57 | 50 | 43 | 52 | 47.1 |
| Multiple bilobar metastases | 50 | 55 | 36 | 33 | 50 | 27 | 39.2 |

**Table 4** Mean number of partial liver resections per hospital per year in 13 Western countries

| *Survey* | *Country* | | | | | | |
|---|---|---|---|---|---|---|---|
| | *France* | *Germany* | *The Netherlands* | *UK* | *USA and Canada* | *Other* | *All* |
| 1985 | 3 | 11 | 2 | 2 | 8 | 6 | 5.4 |
| 1992 | 21 | 28 | 3 | 4 | 15 | 7 | 11.5 |

increased (Table 4). Hospitals in the UK and the Netherlands are less in favor of liver surgery, in contrast to hospitals in Germany, France, the USA and Canada, which employ a more aggressive approach towards liver metastases.

### *Adjuvant chemotherapy*

The third part of the questionnaire surveys adjuvant chemotherapy after resection of a primary tumor or a recurrence. In 79 hospitals (46%) adjuvant chemotherapy was administered after a primary tumor resection for cure. Fluorouracil (5-FU) was administered in 66 (84%) of the hospitals using adjuvant chemotherapy. Therefore it was the most frequently employed chemotherapeutic drug given alone or combined with other drugs. Combinations of 5-FU and levamisole were used in 34 (43%) of the hospitals, and combinations of 5-FU and folinic acid (leucovorin, citrovorum factor) in 24 (30%). Other chemotherapeutic drugs used were doxorubicin (Adriamycin, ADR), mitomycin (MMC), cisplatin (CDDP), methotrexate (MTX), fluorodeoxyuridine (FUdR), methylcyclohexylchloroethylnitrosurea (methyl-CCNU), and interferon. In 62 (78%) of the hospitals systemic administration was used to deliver adjuvant drugs to the patient. Hepatic arterial infusion was employed by 4 (5%) and portal venous infusion by 5 (6%) of the hospitals.

Adjuvant chemotherapy after resection for cure of liver metastases was used in 49 (34%) of the hospitals which performed partial liver resections in 1991. Administrations of 5-FU with or without other chemotherapeutic drugs were used in 38 (78%) of the hospitals. The most frequently used combinations of 5-FU were with levamisole in 11 (22%) and folinic acid in 14 (29%) of the hospitals. Other chemotherapeutic drugs used were doxorubicin, farmorubicin, mitomycin, cisplatin, methotrexate, fluorodeoxyuridine, interferon, and interleukin 2. Systematic administrations were used by 30 (61%), hepatic arterial infusion by 14 (29%) and portal venous infusion by 2 (4%) of the hospitals.

Compared to the 1985 survey the overall use of adjuvant chemotherapy has not changed in Western countries (Table 5). Adjuvant therapy was used less in Germany and the Netherlands, in contrast to France and the UK where its use has increased.

### *Chemotherapy of unresectable liver metastases*

In the fourth and final part of the questionnaire we asked about the use of chemotherapy in patients with unresectable liver metastases. In the 144 hospitals mentioned earlier 2318 (71%) patients had unresectable liver metastases, of which 1172 (51%) were treated with chemotherapy.

The number of hospitals using chemotherapy has increased slightly compared to the 1985 survey (Table 6). This is mainly caused by an increase in the use of chemotherapy in France and the UK. The average number of patients treated with chemotherapy per hospital, however, has dropped from 18 to 12 per year (Table 7). In addition, a shift from local adminisatration towards systemic administration of chemotherapy was observed.

**Table 5** Adjuvant chemotherapy after partial liver resections for cure in 13 Western countries

| *Adjuvant chemotherapy* | *Percentage of hospitals per country* | | | | | | |
|---|---|---|---|---|---|---|---|
| | *France* | *Germany* | *The Netherlands* | *UK* | *USA and Canada* | *Other* | *All* |
| 1985 Survey | 24 | 47 | 9 | 9 | 47 | 44 | 32.3 |
| 1992 Survey | 50 | 20 | 0 | 25 | 44* | 34 | 31.8 |

**Table 6** Chemotherapy of patients with unresectable liver metastases in 13 Western countries

| *Chemotherapy* | *Percentage of hospitals per country* | | | | | | |
|---|---|---|---|---|---|---|---|
| | *France* | *Germany* | *The Netherlands* | *UK* | *USA and Canada* | *Other* | *All* |
| *1985 Survey* | | | | | | | |
| Systemic | 4 | 11 | 13 | 15 | 15 | 12 | 13.0 |
| Local | 36 | 53 | 15 | 21 | 43 | 42 | 33.8 |
| Both | 8 | 37 | 16 | 6 | 33 | 24 | 22.9 |
| Other | 4 | 0 | 3 | 3 | 6 | 6 | 4.2 |
| Total | 52 | 100 | 48 | 44 | 97 | 85 | 73.9 |
| *1992 Survey* | | | | | | | |
| Systemic | 25 | 9 | 36 | 58 | 43 | 42 | 39.2 |
| Local | 0 | 9 | 7 | 8 | 14 | 12 | 10.8 |
| Both | 50 | 64 | 14 | 0 | 21 | 18 | 22.5 |
| Other | 25 | 0 | 0 | 17 | 0 | 9 | 5.9 |
| Total | 100 | 82 | 57 | 83 | 79 | 82 | 78.4 |

**Table 7** Mean number of patients with unresectable liver metastases treated with chemotherapy per hospital per year in 13 Western countries

| *Survey* | *Country* | | | | | | |
|---|---|---|---|---|---|---|---|
| | *France* | *Germany* | *The Netherlands* | *UK* | *USA and Canada* | *Other* | *All* |
| 1985 | 15 | 34 | 10 | 12 | 20 | 15 | 18.0 |
| 1992 | 12 | 17 | 5 | 7 | 20 | 8 | 11.6 |

### DISCUSSION

The present study was undertaken as a sequel to a postal survey on attitudes toward detection and management of hepatic metastases held in 1985[3]. In contrast to the 1985 survey, the recent survey had a low response rate of only 21%. Several factors may have contributed to this low response rate: (1) The extent of the questionnaire. The recent questionnaire not only contained more questions, but also asked more detailed questions and assumed that data on the number of patients with partial liver resections and chemotherapy were available. (2) Satiety. Two other postal surveys closely related to ours were held in 1992[4,5]. (3) Incorrect addresses. In the recent survey, using the 1990 HPB membership registry, at least 3% of the addresses were incorrect, compared to zero percent in 1985 survey.

Because only 171 questionnaires were returned to us, we decided against the use of inferential statistical techniques. Therefore, only observed frequencies are reported in this paper. Also, we feel it is inappropriate to compare the 1985 and the recent surveys using inferential statistics, because of the possible differences between the two groups of respondents. The results of the recent survey, however, could be of value in the light of a worldwide discussion on colorectal cancer follow-up, with special reference to the detection and resection of hepatic metastases.

Our survey indicated that systematic routine follow-up of colorectal cancer patients operated on with curative intent was used by 88% of the hospitals. The majority of these hospitals used follow-up schemes of five years or more. Scheduled history taking and physical examinations, routine liver biochemistry and carcinoembryonic antigen (CEA) monitoring were the most popular and frequently used tests. Although there is a National Institutes of Health (NIH) consensus statement on the use of CEA [6], there still remains controversy about the diagnostic and therapeutic policy following an unexplained rise in CEA. Only 37% of the hospitals using CEA were willing to perform second-look laparotomies based on significant and consecutive rises of CEA-levels only, as advocated by Minton *et al.*[7] Alternatively, many hospitals may have responded to such a rise by increasing the intensity of follow-up.

The percentage of hospitals resecting liver metastases in the 1992 survey has increased only slightly compared to the 1985 survey. The number of resections per hospital, however, has doubled from 6 to 12 resections per year. Also, the extent of liver operations has increased. In the 1985 survey mainly solitary and multiple unilobar liver metastases were resected, compared to multiple unilobar and bilobar metastases in the 1992 survey. The majority of the hospitals (79%) regarded patients with more than 3 or 4 hepatic metastases in the absence of extrahepatic disease as unresectable. This observation may give cause for concern. Since the introduction of more sensitive imaging techniques in recent years, smaller hepatic metastases can be identified preoperatively. Approximately 70% of the hospitals used extremely sensitive imaging techniques like CT-angiography, CT-portography, or MRI. The use of these recently developed techniques would classify more patients as unresectable. Scheele[8] proposed that resection to benefit prognosis seems justified as long as complete tumor clearance can be achieved, independent of number, distribution or size of liver metastases. Therefore, the decision to resect hepatic metastases should depend on the sensitivity of the preoperative imaging techniques used, and not on the application of maximum numbers found in the literature [9, 10]. Intraoperative ultrasound should be used to assess the resectability during the operation. The additional value of this technique, however, remains hypothetical. In our survey no increase in the percentage of unresectable patients during operation was observed when ultrasonography was used.

In less than half of the hospitals (46%) adjuvant chemotherapy was administered following primary tumor resection for cure. Adjuvant chemotherapy after resection of hepatic metastases was used in 34% of the hospitals which performed liver surgery. This may indicate that the use of adjuvant chemotherapy is still not very popular. Combinations of fluorouracil (5-FU) and levamisole with or without folinic acid (leucovorin, citrovorum factor) were most frequently used, conforming to the NIH consensus statement on adjuvant chemotherapy for colorectal cancer patients[2]. The majority of the hospitals used adjuvant chemotherapy only when they collaborated in a clinical trial.

Patients with unresectable liver metastases received chemotherapy in 78% of the hospitals. Over the last years local chemotherapy has become less popular, while systemic chemotherapy has gained in popularity.

Hospitals in the UK and the Netherlands make use of less intensive follow-up compared with other Western countries. Although the percentage of hospitals in favor of liver surgery has increased according to our recent survey in these two countries, a less aggressive surgical attitude was found. Also per hospital less patients were operated for liver metastases compared to other countries. These findings are supported by the results of postal surveys of Foster *et al.*[11] and Karanjina *et al.*[12] in England and Wales.

The results of our survey on the attitudes towards detection and management of hepatic metastases of colorectal origin indicate that there is no consensus on the follow-up of colorectal cancer patients. The most frequently applied diagnostic test in colorectal cancer follow-up was serial CEA determinations. However, the frequency of these determinations in the first years after primary tumor resection was low in most hospitals. Also, unexplained rises of CEA-levels were not followed by second-look laparotomies in the majority of the hospitals. Therefore, the current use of CEA-testing is questionable. Another matter on which no agreement is found, is the maximum number of liver metastases justifying partial liver resection. There may be an increasing awareness that resection criteria should not be limited to a fixed number metastases, but should be based on the probability of ensuring free surgical margins. Finally, there is consensus on the use and type of adjuvant chemotherapy in colorectal cancer patients. However, most hospitals do not offer this on a routine basis to their patients.

In conclusion, this survey indicates that there is much need for prospective randomized trials on which worldwide consensus can be reached regarding detection and management of hepatic metastases of colorectal origin.

### ACKNOWLEDGEMENTS

The authors wish to thank all persons who responded to the questionnaire.

### REFERENCES

1. Boring, C. C., Squires, T. S., Tong, T. (1993) Cancer statistics. *CA*, **43**, 7–26.
2. NIH consensus conference (1990). Adjuvant therapy for patients with colon and rectal cancer. *JAMA*, **264**, 1444–1450.
3. van de Velde, C. J. H., de Brauw, L. M. (1988) Attitudes towards detection and management of hepatic metastases in the Western world. *Eur. J. Cancer Clin. Oncol.*, **24**, 791–794.
4. Vidal-Jove, J., Sugarbaker, P. H. (1991) Repeated resection of colorectal hepatic metastases [questionnaire]. Washington DC: The Cancer Institute, Washington Hospital Center.
5. Wade, T. P., Johnson, F. E. (1992) Practice patterns of postoperative follow-up care of patients following treatment of colon carcinoma [questionnaire]. St. Louis (MO): Saint Louis University Medical Center.
6. Summary of an NIH consensus statement (1981). Carcinoembryonic antigen: Its role as a marker in the management of cancer. *Br. Med. J.*, **282**, 373–375.
7. Minton, J. P., Hoehn, J. L., Gerber, D. M. *et al.* (1985) Results of a 400-patient crcinoembryonic antigen second-look colorectal cancer study. *Cancer*, **55**, 1284–1290.
8. Scheele, J. (1993) Hepatectomy for liver metastases. *Br. J. Surg.* **80**, 274–276.
9. Adson, M. A. (1987) Resection of liver metastases—When is it worthwhile? *World J. Surg.*, **11**, 511–520.
10. Registry of Hepatic Metastases (1988). Resection of the liver for colorectal carcinoma metastases: A multi-institutional study of indications for resection. *Surgery*, **103**, 278–288.
11. Foster, M. E., Hill, J., Leaper, D. J. (1987) Follow-up after colorectal cancer—current practices in Wales and South West England. *Int. J. Colorect Dis.* **2**, 118–119.
12. Karanjina, N. D., Rees, M., Schache, D., Heald, R. J. (1990) Hepatic resection for colorectal secondaries. *Br. J. Surg.* **77**, 27–29.

### APPENDIX

### 2nd QUESTIONNAIRE ON LIVER METASTASES TREATMENT POLICY OF COLORECTAL ORIGIN

### I Detection

1. Did your department use a **SYSTEMATIC** follow-up aimed at detecting liver metastases in patients operated with curative intent for colorectal cancer in 1991? If yes, please mark the appropriate boxes in the table below. ☐ Yes ☐ No

| Months | 3 | 6 | 9 | **12** | 15 | 18 | 21 | **24** | 27 | 30 | 33 | **36** | 39 | 42 | 45 | **48** | 51 | 54 | 57 | **60** |
|---|---|---|---|---|---|---|---|---|---|---|---|---|---|---|---|---|---|---|---|---|
| Clinical Evaluation | | | | | | | | | | | | | | | | | | | | |
| Routine Liver Biochemistry | | | | | | | | | | | | | | | | | | | | |
| Liver Ultrasound | | | | | | | | | | | | | | | | | | | | |
| Liver CT | | | | | | | | | | | | | | | | | | | | |

2. Was **CEA ASSAY** also part of a systematic follow-up aimed at detecting liver metastases in 1991? ☐ Yes ☐ No
If yes, please specify the frequencies of CEA assaysin 1st, 2nd, 3rd, 4th, and 5th year
........................................................................................
........................................................................................

3. Are you willing to perform second look laparotomy on the basis of elevated **CEA ONLY** (without any additional evidence on the presence of liver metastases by other diagnostic tests)? ☐ Yes ☐ No

4. Did you use **OTHER TUMOUR MARKERS** in the systematic follow-up aimed at detecting liver metastases in 1991? ☐ Yes ☐ No
If yes, which tumour markers did you use?
........................................................................................
Please specify the frequencies of tumour marker detections in 1st, 2nd, 3rd, 4th, and 5th year.
........................................................................................
........................................................................................

### II Surgery

1. In how many patients were liver metastases detected in 1991? .... pts.

2. If **POTENTIALLY** resectable liver metastases were detected, what were your criteria for considering surgery in 1991?
a. Never consider surgery (⇒ continue with section III) ☐ a.
b. One solitary metastasis only ☐ b.
c. Multiple metastases confined to one lobe (please specify how many) ☐ c. ....
d. Multiple metastases not confined to one lobe (please specify how many) ☐ d. ....

3. When you suspected the presence of liver metastases, by what means did you **PRE-OPERATIVELY** assess their resectability? (more than one item is allowed)
a. Ultrasound Imaging ☐ a.
b. Computerized Tomography ☐ b.
c. Computerized Tomography with Angiography ☐ c.
d. Magnetic Resonance Imaging ☐ d.
e. Scintigraphy ☐ e.

4. How many patients underwent surgery for potentially resectable liver metastases in 1991? .... pts.

5. In how many of these patients were liver metastases actually resected? .... pts.

6. Did you **INTRAOPERATIVELY** assess the resectability of liver metastases by ultrasound imaging?
   ☐ Yes
   ☐ No

### III Adjuvant Chemotherapy

1. Following potentially curative resection for **COLORECTAL CARCINOMA**, was any **ADJUVANT** chemotherapeutic treatment given routinely in 1991?
   ☐ Yes
   ☐ No
   If yes, which drugs did you administer, and by which route?
   ..........................................................................................

2. Following potentially curative partial **LIVER** resection, was any **ADJUVANT** chemotherapeutic treatment given routinely in 1991?
   ☐ Yes
   ☐ No
   If yes, which drugs did you administer, and by which route?
   ..........................................................................................

### IV Chemotherapy

1. In how many patients were **IRRESECTABLE** liver metastases detected in 1991? .... pts.

2. If irresectable liver metastases were detected, what was your policy regarding chemotherapy in 1991?
   a. No further treatment ☐ a.
   b. Systemic I.V. chemotherapy ☐ b.
   c. Local (intra-arterial or intraportal) chemotherapy ☐ c.
   d. Other treatment (please specify) ☐ d.
   ..........................................................................................

3. How many of these patients were actually treated with chemotherapy in 1991? .... pts.

